# Advanced Injection Molding Methods: Review

**DOI:** 10.3390/ma16175802

**Published:** 2023-08-24

**Authors:** Mateusz Czepiel, Magdalena Bańkosz, Agnieszka Sobczak-Kupiec

**Affiliations:** Department of Materials Engineering, Faculty of Materials Engineering and Physics, Cracow University of Technology, 37 Jana Pawła II Av., 31-864 Krakow, Poland; mateusz.czepiel1@doktorant.pk.edu.pl (M.C.); agnieszka.sobczak-kupiec@pk.edu.pl (A.S.-K.)

**Keywords:** injection molding, plastics, processing methods, advanced molding technology

## Abstract

Injection molding is a method commonly used to manufacture plastic products. This technology makes it possible to obtain products of specially designed shape and size. In addition, the developed mold allows for repeated and repeatable production of selected plastic parts. Over the years, this technology grew in importance, and nowadays, products produced by injection molding are used in almost every field of industry. This paper is a review and provides information on recent research reports in the field of modern injection molding techniques. Selected plastics most commonly processed by this technique are discussed. Next, the chosen types of this technique are presented, along with a discussion of the parameters that affect performance and process flow. Depending on the proposed method, the influence of various factors on the quality and yield of the obtained products was analyzed. Nowadays, the link between these two properties is extremely important. The work presented in the article refers to research aimed at modifying injection molding methods enabling high product quality with high productivity at the same time. An important role is also played by lowering production costs and reducing the negative impact on the environment. The review discusses modern injection molding technologies, the development of which is constantly progressing. Finally, the impact of the technology on the ecological environment is discussed and the perspectives of the process were presented.

## 1. Introduction

**Figure 1:**
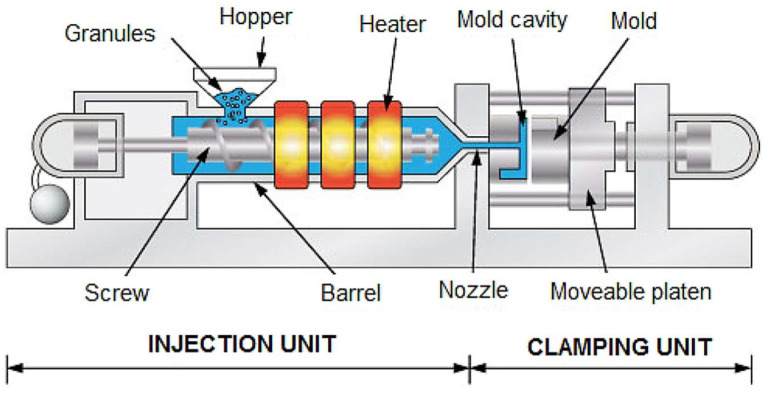
Scheme of injection molding machine [22].

Injection molding technology is one of the most common methods used in the plastics processing industry. It is a method that can be used for mass production of products with complex shapes [1,2]. It is used in many industries, not only in the production of children’s toys and medical equipment, but also in the automotive or aerospace industries. Injection molding technology is a method to obtain molded products by injection molding of plastic [3,4,5]. The plastic is melted under heat and injected into a suitable mold and then cooled and solidified. Injection molding was known for many years. However, the process was refined over time. Different variations of injection molding technology were developed in response to new challenges in technology and new types of plastics. The need for change is also driven by the increasing expectations of manufacturers in various industries for the final products, as well as the target customers of these products [6,7]. The first work on plastic injection molding dates back to the 19th century. Initially, a relatively simple machine was developed to mold buttons, hair combs, and other small items. In the 20th century, developments in polymer chemistry were instrumental in introducing new plastics, such as polyethylene, polypropylene, and polystyrene, which were ideal for injection molding. Work intensified with the development of the first practical injection molding machine in 1946, which made it possible to automatically inject plastic into a mold. This invention also contributed to the development of injection molding technology and greatly facilitated the previously known process [8,9,10]. In the following years, injection molding became widely used in industry. The main focus was on designing equipment with greater power and with automation of the process. The development of advanced molding techniques contributed to increasing the efficiency and precision of this production method. With the development of technology in the field of polymeric materials, injection molding found application in an increasing number of industries [11,12]. From automobiles to electronics, injection molding became one of the most important manufacturing techniques [13]. Injection molding technology involves cyclic plasticization of plastic under a high temperature (Figure 1). The melted plastic is then injected under a certain pressure into a mold cavity, in which, after solidification, a specific product shape is given [14,15,16]. The first step is proper preparation of the mold by mapping the final shape that the finished product is to have. Since it is possible to obtain virtually any shape of the product and the mold must be properly designed for each component separately, this technology is cost-effective primarily for high-volume production [17,18]. The plastic is usually used in powder or pellet form and is subjected to high temperatures to melt and plasticize it. Further, the plastic mass under high pressure is injected through an injection molding machine system. Injection must be rapid and at the right temperature of the plastic and the mold so that it does not “freeze” during the filling phase, which may cause underfilling. After the entire mold is filled with the melted plastic, the whole is cooled so that the product obtains the specified shape. The time of cooling depends on the type of plastic and the thickness of the product. Once the finished product is obtained, additional processing may be required, including removal of excess plastic, painting, or assembly of several parts [19]. Injection molding plastic processing has several important advantages, most importantly, the mentioned flexibility of the molded product shape and size. However, the size of the detail is limited by the size of the injection molding machine, and equally important are specific rules in the design of the mold to allow the removal of the molded product after processing. In the case of small parts, often during one molding cycle, several or a dozen parts are made simultaneously, which affects the speed of the process. Thus, this method allows for economical production of large batches of products. In addition, injection molding is a popular process due to its high precision, speed of production, repeatability, and possibility of mass production [20,21].

The paper discusses the most commonly processed plastics by injection molding. It also focuses on literature reports describing the influence of process parameters on its performance and the final properties of products. In addition, interesting research work carried out in various types of injection molding is discussed; in particular: water-assisted injection molding; gas-assisted injection molding; microcellular injection molding; variable mold temperature technologies; microinjection molding; and rapid thermal cycling molding. The impact of the process on the ecological environment is further discussed and prospects for further development are presented.

## 2. Materials Used in Injection Molding

### 2.1. Polypropylene

Polypropylene belongs to the polyolefin group and is one of the most widely used plastics in injection molding [23]. The advantage of using this plastic is its low viscosity in the molten state, which makes it possible to obtain a smooth and fluid consistency. It is easily moldable and allows for the molding of products of any shape. It is a material resistant to erosion, rust, and chemical spills [24,25,26]. In addition, it is considered a material with a relatively low price and high availability. Polypropylene is widely used in industry in the production of packaging and household products, but also in the automotive and electronics industries, as well as in the manufacture of sporting goods and children’s toys. This material is used to produce, among others, tanks, medical equipment, syringes, technical textiles, carpets, enclosures for electrical appliances, wires, cables, car parts, bathroom fittings, and household goods [27]. In injection molding technology, different varieties of polypropylene or its blends with other polymers or modifying additives can be used. The selection of the appropriate material depends on the desired properties of the material to be produced [28,29].

**Figure 2:**
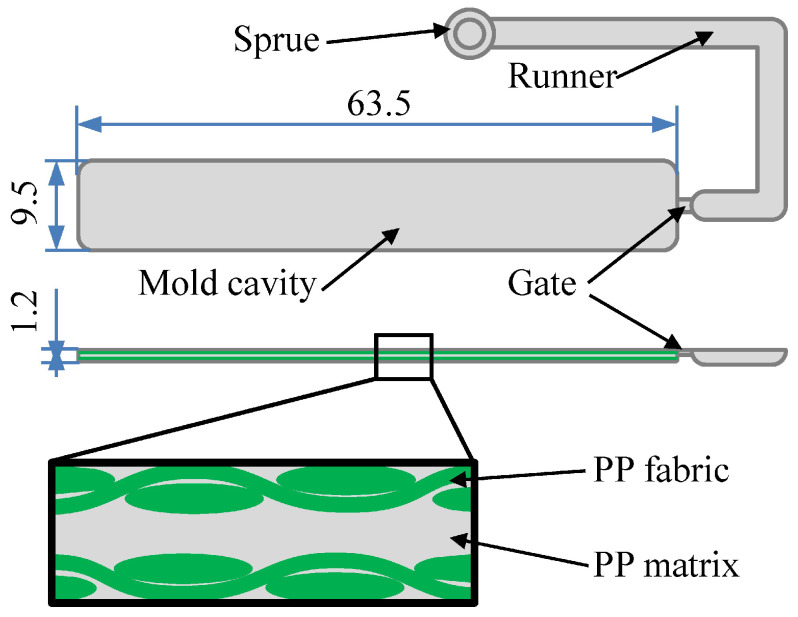
Schematic of the mold cavity and injection molding structure of the PP SPC sample insert (unit on the figure mm) [32].

Research on the use of polypropylene in injection molding techniques was presented by Farotti et al. The paper presents the results of a study of the mechanical characteristics of commercial PP. The analysis was aimed at determining the correlation between the input injection molding parameters and the mechanical behavior of the material. Particular attention was paid to determining the influence of parameters such as plastic melting point, mold temperature, injection pressure, and cooling time. Based on the analysis, the influence of mold temperature and injection pressure on the mechanical properties of the polymer was confirmed. Increasing the values of these factors can lead, among other things, to distortion of the obtained product [30]. An interesting study also relates to the results presented by Andrzejewski et al. The aim of the study was to compare two types of polymer fillers, which are used during polypropylene processing by injection molding. The possibility of using buckwheat hulls as an alternative to wood fiber was tested. The potential of this filler in polypropylene processing technology was confirmed [31]. The injection molding process of single-polymer composite (SPC) products using PP was presented by Wang et al. It was found that the sample weight and tensile properties of the PP SPCs varied in different rules with the variations of these four parameters. Significant effects were determined for cylinder temperature, injection pressure, and also holding time. A diagram of the mold cavity and injection molding structure of the PP SPC sample insert is presented in Figure 2 [32].

Kosciuszko et al. then proposed an analysis of the change in the value of re-molding shrinkage and voids formed in PP moldings. The study was carried out by determining the dependence on the degree of porosity and time after removal of the molded part from the injection mold cavity. It was proven that the cell injection process, together with a longer holding phase, allows the reduction in gas pores, which translates into the reduction in cavities and unevenness of products [33].

### 2.2. Polyethylene

Polyethylene is one of the most popular and widely used polymers. It is obtained by polycondensation of ethylene [34]. Due to the complex structural hierarchy, there are three main types of polyethylene, such as low-density PE (LDPE), high-density PE (HDPE), and linear low-density PE (LLD-PE). Each variety differs in terms of molecular weight and chain branching [35,36]. Polyethylene is often used because of its properties. It is considered a polymer that is easy to process and has relatively good mechanical properties. In addition, it is characterized by flexibility and ductility as well as resistance to heat, electricity, chemical agents, and degradation. Polyethylene is widely used in many fields, including packaging, construction, agriculture, medicine, automotive, and many others. Its versatile properties make it an extremely useful material in many areas of everyday life [37,38].

Due to its plasticity and ease of processing, one of the most popular ways of producing PE products is injection molding. The molten polymer is injected into an injection mold and then cooled and hardened to obtain the desired shape. The injection molding method can be used to obtain many different types of products from this plastic. Among other things, it is used to produce various types of packaging, such as bottles and boxes. It is also widely used in the production of medical products, such as syringes, but also in the automotive industry and in household goods. In addition, many toys are obtained from PE through the use of injection molding [39,40,41].

PE processing by injection molding is the subject of many scientific studies. Leyva-Porras et al. were concerned with determining the effects of processing variable parameters on the microstructure and crystallinity of LDPE samples that were injection molded. The polymer was injected at different cylinder and mold temperatures. It was proven that the interaction of the two temperatures has the greatest effect on the size of the spherulite. In turn, the mold temperature has a significant effect on crystallinity [42]. Subsequently, Meszaros et al. conducted research on a self-reinforcing composite material that can be easily processed by injection molding. Again, polyethylene was chosen as the base material [43]. Kumar Lal et al. studied issues related to PE shrinkage after removal from the mold. The shrinkage of the polymer is one of the most important issues that affect the change in dimension during the injection molding process. The purpose of this study was to develop optimal parameters for injection molding of low-density polyethylene. It was found that minimum shrinkage of LDPE was obtained at a melting temperature of 190 °C, injection pressure of 55 MPa, filling pressure of 85 MPa, and cooling time of 11 s. The factor of greatest importance was found to be the cooling time. In contrast, injection pressure was the least effective parameter [44]. Khan et al. then presented a parameter optimization design for recycled HDPE products. They used Gray’s relational analysis to determine the optimal combination of injection molding parameters. The results indicate that the best set of parameters for recycled HDPE products is as follows: melting temperature 240 °C, clamping pressure 255 N/m^2^, injection time 0.6 s, and holding time 30 s [45]. In contrast, Djurner et al. determined the effect of injection pressure on two types of low and high molecular weight PE. Based on their analysis, the researchers concluded that the use of high pressures during HDPE injection molding leads to a material with desirable mechanical properties [46].

### 2.3. Polystyrene

**Figure 3:**
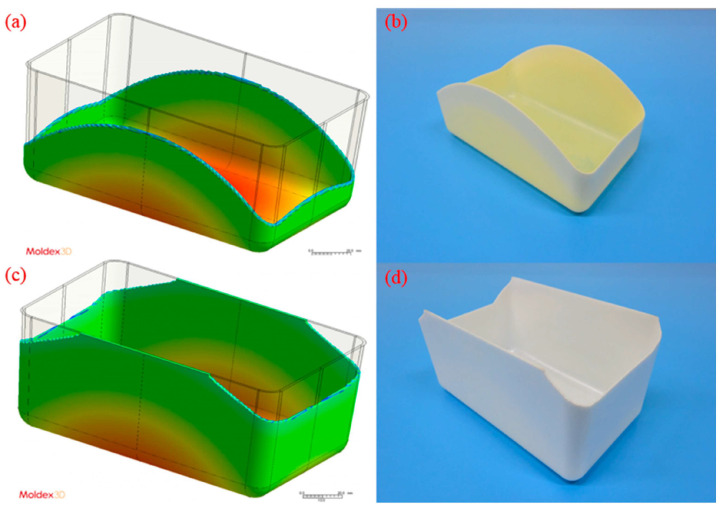
Simulated and experimental results using medium-level process parameters (i.e., 100 cm^3^/s injection speed, 210 °C melt temperature, and 60 °C mold temperature): (**a**) simulated result for the PP sample, (**b**) actual experimental result for the PP sample, (**c**) simulated result for the PS sample, and (**d**) actual experimental result for the PS sample [58].

Polystyrene is a plastic obtained by polymerizing styrene, and belongs to the polyolefin group [47,48]. This polymer is widely used in industry as an insulating and packaging material, as well as in the production of various consumer goods [49]. Polystyrene is used for the production of, among others, acid-resistant pipes, electrical components, insulators, household goods, car parts, toys, packaging, haberdashery and everyday products, and foam thermal insulation materials used in construction and refrigeration (e.g., polystyrene) [50]. It is one of the most widely used polymers in the world. Polystyrene can come in various forms, but it is most commonly found as plastic or as expanded polystyrene (EPS), also known as polystyrene. In its natural state, this polymer is transparent with a high surface gloss. PS is included in the group of thermoplastics, or materials whose shape can easily be given during the melting process [51,52]. PS exhibits good electrical and dielectric properties. It is resistant to moisture, some salts and acids, and also to abrasion. However, it is flammable and has low heat resistance [53]. It is processed mainly by injection molding and is another material widely used in this type of processing. Due to its high ductility and ease of molding, it can be customized into various shapes and sizes. Considering the injection molding process, it is worth noting that polystyrene has excellent processing properties that make it easy to mold by injection molding. It has a low melting point, which means it requires lower processing temperatures and shorter injection molding cycles [54,55]. However, many parameters determine the quality of the final product and the efficiency of the process. Among other things, mold coatings were evaluated to reduce the melt flow resistance of polystyrene through the injection molding machine [56]. The properties of injection molding PS additionally filled with carbon fiber to increase the mechanical strength of the resulting products were also investigated [57]. A comparative analysis of semi-crystalline PP and amorphous PS was also carried out. It was shown that semi-crystalline materials foamed less due to crystallinity. Amorphous PS, on the other hand, showed the highest expansion coefficient at high injection speed vs. low mold temperature [58]. The results of this experiment are presented below in Figure 3.

Subsequently, SadAbdai et al. proposed work on the numerical calculation of the fiber orientation tensor of an injection-molded short glass fiber polystyrene (SGF-PS) composite part, shaped as a rectangular plate. Again, the effect of selected injection parameters, such as mold wall temperature, injection flow rate, and also fiber content on the change in orientation of this sample was checked. Based on simulations, the injection flow rate was found to have a greater effect on the fiber orientation than the mold wall temperature [59]. Polystyrene was also studied in high-pressure foam injection molding. It was shown that the cell structure of the resulting foams can be controlled by selecting appropriate injection molding process parameters [60].

### 2.4. Acrylonitrile-Butadiene-Styrene

**Figure 4:**
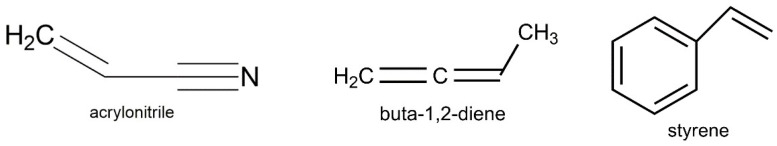
ABS copolymer components.

Acrylonitrile-butadiene-styrene copolymer (ABS) is an amorphous polymer obtained by emulsion polymerization or bulk polymerization of acrylonitrile with styrene in the presence of polybutadiene [61,62]. This copolymer consists of 15–35% acrylonitrile, 5–30% butadiene, and 40–60% styrene. The components of the copolymer are presented in Figure 4. The characteristics of this material are high impact strength, impact resistance, and hardness. In view of this, ABS is often used for the manufacture of covers or housings for components that require protection from mechanical damage. In addition, ABS has good insulating properties, high dimensional stability, and low moisture absorption [63,64,65]. ABS is most commonly used for the manufacture of enclosures of electrical appliances, electronic devices, automotive components (such as car trusses, wheel arches, and some body parts) [66].

ABS is a material eagerly used in injection molding [67]. Many studies were conducted to optimize the process taking into account the properties of this plastic. Linear models of the mechanical properties of ABS were determined using the Taguchi method, and through analysis of variance, the effect of varying process parameters was determined. Relationships between injection parameters and mechanical properties of ABS moldings, e.g., elastic modulus, tensile strength, bending modulus, and impact strength, were determined [68]. Next, the re-injection processing capability of two types of ABS with low u high viscosity was evaluated. Experimental results indicate the significant qualitatively different behavior of the polymers after reprocessing. As the number of reprocessing cycles increased, a decrease in viscosity was noted for the low initial viscosity polymer. Conversely, for a high-viscosity polymer, increasing the number of injection molding cycles led to an increase in viscosity [69]. The feasibility of ABS reprocessing was also investigated by Rahimi et al. They determined the mechanical properties of the polymer through five-stage reprocessing [70]. Research was also conducted to determine the energy intensity of the ABS injection molding process. For this purpose, direct measurement of equipment energy and analysis of molding quality were carried out. The parameter of polymer holding time and mold cooling time played the greatest role in energy consumption [71]. Volpato et al. then conducted a comparative analysis of the properties of steel and ABS moldings [72]. Sreedharan et al. determined the effect of injection pressure and cooling systems on the degree of shrinkage of ABS copolymer products as automotive components [73]. On the other hand, Lay et al. compared the processing of ABS fused deposition modeling (FDM) and conventional injection molding [74]. Subsequently, surface properties were also determined in automotive interior components produced by ABS injection molding. They discussed the correlations between process parameters and the measured gloss and surface properties of the resulting products [75].

### 2.5. Polyvinyl Chloride

Polyvinyl chloride (PVC) is one of the most popular and widely used synthetic polymers. It is a thermoplastic polymer that consists of repeating units of vinyl chloride [76] PVC is widely used in the construction industry, where it is commonly used to make pipes and fittings. Thanks to its corrosion resistance, durability, and ease of processing, PVC is the material of choice for creating sewer systems, water systems, and pipes for conducting various types of substances. It is also used in the manufacture of windows and doors, as well as widely in the packaging industry. In addition, PVC is used in the furniture, medical, and automotive industries [77,78]. PVC fittings obtained by injection molding were studied by Llado et al. The effect of injection molding parameters on the formation of efflorescence on the surface of the parts obtained was determined. It was determined that the main reason for the formation of defects was an incorrectly selected injection rate. Melt temperature was identified as the second factor of importance [79]. The identification of parameters affecting the formation of warpage of PVC moldings was also addressed by Ahmed et al. A mathematical model was developed to regulate warpage before production and minimize losses [80]. Subsequently, Tsai et al., using Moldex3D and the Taguchi method, determined the possibility of eliminating imbalanced filling of multicavity molds for PVC injection molding [81].

### 2.6. Polymethyl Methacrylate

Polymethyl methacrylate, also known as acrylic glass, is a thermoplastic artificial polymer with a transparent and glass-like structure. It is one of the most widely used polymers due to its unique properties, such as high transparency, UV resistance, mechanical strength, and ease of processing [82]. It is used extensively in optical products, lighting products, and electronic housings. It plays an important role in orthodontics and dentistry [83]. PMMA can be successfully used in obtaining products by injection molding methods. Two-component products of polycarbonate and polymethylmethacrylate were obtained [84]. PMMA was then used for injection molding in a nanotube with an aspect ratio of 2.0. The mechanism of residual stresses was determined using molecular dynamics simulations [85]. Additionally, Zhang et al. identified residual stresses as having great significance in product quality and microstructure properties. PMMA was used to develop an injection molding simulation model for determining the effect of processing parameters on residual stresses [86]. Subsequently, it was shown that injection-molded PMMA can have worse mechanical properties (impact strength and bending strength) than thermally cured PMMA [87]. However, it is possible to combine PMMA with other additives controlling the properties of the resulting products [88].

### 2.7. Polyamide

Polyamide, also known as nylon, is one of the most important thermoplastic engineering materials used in injection molding. It is a versatile polymer with high mechanical strength, abrasion resistance, low hardness, and well-defined thermal properties. Polyamide was repeatedly analyzed as a material that can be processed by injection molding methods [89,90]. The properties of a composite molding of polyamide 6.6 reinforced with long glass fibers were determined [91]. The possibility of processing this polymer together with natural Curauá cellulose fibers [92] was tested. The microstructure and mechanical properties of injection-molded microporous nanocomposites of polyamide-6 and nanoclay [93] were determined. Attempts were made to combine this polymer together with polypropylene and nanoclay [94,95]. In addition, studies were conducted to determine the correlation between the cell size of microstructured nanocomposites with PA and tensile strength [96].

## 3. Injection Molding Techniques

### 3.1. Water-Assisted Injection Molding

**Figure 5:**
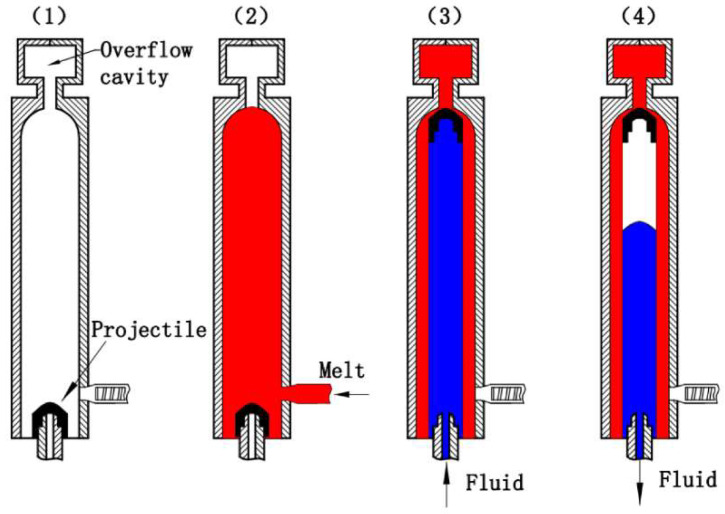
Forming process of the F-PAIM overflow method process [98].

Polymer processing technologies developed significantly in recent years. One of the new methods is water-assisted injection molding (WAIM). WAIM technology is proving to be a promising technology due to the lightness of the products produced, shorter cycle times, and greater potential for hollow product production. Water- or gas-assisted technologies can be categorized as F-PAIM or fluid projectile-assisted injection molding methods. The F-PAIM process can be divided into two types: the short-shot method and the overflow method. These methods differ depending on whether the mold cavity is filled with molten polymer completely or partially [97]. Figure 5 shows a diagram of the overflow method. In this case, the cavity is filled completely with polymer. First, the bullet is placed on the liquid nozzle. Then the mold is filled completely with molten polymer. Next, the fluid is injected under pressure. A cavity is produced, which is maintained by the fluid pressure compensating for any shrinkage. Finally, the system is cooled and the fluid is drained even before the mold is opened. Summary: (1) To begin with, the projectile is positioned onto the fluid nozzle. (2) Next, the mold is closed, and the molten material is injected into the mold cavity, covering the projectile until the cavity is completely filled with the molten material. (3) Subsequently, pressurized fluid is introduced through the fluid nozzle after a specific gas injection delay time. This pressure propels the projectile through the molten core, leading it into an overflow cavity. (4) Lastly, the component undergoes cooling, while the fluid pressure maintains its shape by compensating for any shrinkage. Once the cooling is complete, the fluid is drained before the mold is opened, allowing the part to be ejected [98].

In the case of the short shot method, the cavity is filled with molten polymer only partially. In the system designed for the WAIM method, an additional water injection unit is distinguished in addition to the conventional system for injection molding. For the most part, this unit consists of a water pump, a water tank equipped with a temperature controller, a pressure accumulator, and automatic circuit control. The control system is responsible for parameters such as the time and pressure of water injection. Usually, such a unit has its own hydraulic system, is mobile, and can be adapted to different forms of machine. Different designs of water pins can be used: immobile pin type [99,100] and movable pins [101], respectively. Research is also being conducted on the use of water pins of different designs, such as ring-type pin and orifice-type pin. After forming the products, the length of water penetration in the moldings was checked depending on the type of pin used. Both the shape of the hollow sprues and the length of the hollow cores were determined by the type of pin used [102]. The phenomenon of water penetration in the process of injection molding of semi-crystalline PP was studied by Ahmadzai et al. Three parameters, such as water penetration delay time, holding time, and mold temperature, were checked. Wall thickness, shrinkage, and degree of homogeneity were checked for the resulting moldings. The desired properties, i.e., a product with low shrinkage and a smaller difference in wall thickness, were obtained using a high mold temperature and a long holding time [103].

The problem with the WAIM method is the phenomenon of “fingering” of water. This occurs when water bubbles get outside the water channels. It was proven that amorphous materials molded by the WAIM method produce less fingering than when using semi-crystalline polymers. The smallest side effects were also obtained for water channels with a rib gouged on top. Water pressure and injection size are also important parameters [104]. The phenomenon of fingering was also described. During the injection molding of composites, worse properties were obtained with fiberglass than with polybutylene terephthalate materials that were not modified [105]. Next, the mechanical properties of water-assisted injection-molded products were also studied. Irregular water penetration in the moldings and the effect of water temperature on the crystallinity of the moldings were found. However, the correlation of bonding with mechanical properties was relatively low [106]. The possibility of modifying the WAIM method to mass-produce tubular parts of high complexity was also tested. Several water injectors, designed in the direction of the corresponding branches, were used simultaneously. The method is designed to reduce production time and provide raw material savings. It offers a cost-effective way to produce large parts with good surface finish, reduced weight, and relatively short cycle times [107]. Studies on the influence of selected parameters on the properties of products obtained by the WAIM method are also described in [108,109,110,111].

### 3.2. Gas-Assisted Injection Molding

Gas-assisted injection molding (GAIM) is an injection molding method in which gas is also injected along with the injected plastic [112,113]. Injecting gas into the mold allows for controlled filling of the molding space and uniform distribution of the plastic. This, in turn, leads to reduced deformation, stress, and shrinkage of the material. Parts molded using this technique have higher dimensional precision and better shape stability [114,115,116]. Example values of processing parameters using the GAIM technique are presented in Table 1.

A significant problem in injection molding is the possibility of scorch marks on parts of finished products. Reducing scorch marks by reducing the clamping pressure can be achieved with gas-assisted technology [118]. The use of gas-assisted technology allows for reduced stresses on molded parts, reduced dropout, and greater design freedom. The GAIM technique was simulated in the manufacture of a plastic disk holder that will allow it to be placed in an optical reader. The simulations made it possible to determine the optimal parameters to ensure a significant reduction in production time and cost [117,119]. The Phan-Thien Tanner (PTT) constitutive model was also presented for further simulations for the GAIM technique. The model assumes consideration of the most important viscoelastic properties [120]. The use of a gas-assisted method for processing eco-composites was also proposed. The use of naturally derived polymers in injection molding has limitations due to the increased shear viscosity of these materials. However, it was demonstrated that the gas-assisted method can be successfully used in such a case; for example, for rice husk-filled poplipropylene-based eco-composite polymers [121]. The gas-assisted technique can also be successfully combined with microcell injection molding. The combination of these two methods enables significant weight reduction, and also improves the surface appearance and mechanical properties of the resulting moldings [122]. Importantly, gas-assisted molding techniques can also be applied to the gas-assisted mold temperature control method [123,124,125].

### 3.3. Microcellular Injection Molding

**Figure 6:**
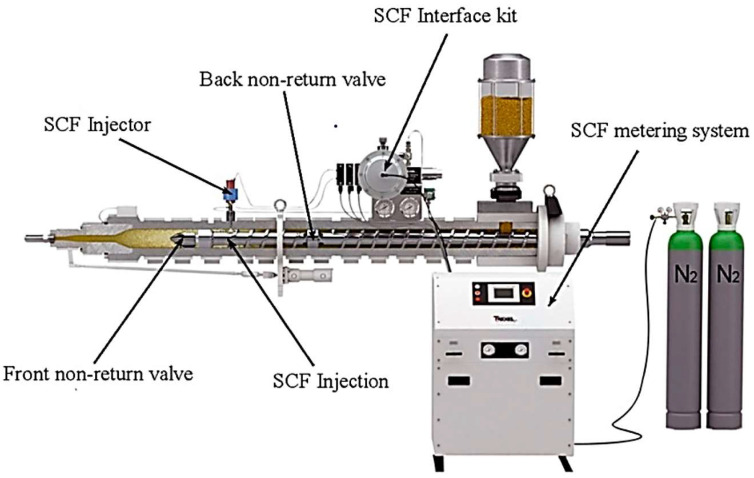
The structure of a typical miclocellural system (MuCell^®^) [126].

Microcellular injection molding (MIM) is a technique that enables the production of lightweight plastic products with a microcellular internal structure. The process in question allows for a material reduction of 30% to 40%. In addition, the resulting products have higher impact strength and an internal structure composed of a high density of small bubbles. In the injection molding process, liquid plastic is injected into the mold under high pressure, and then the pressure is quickly reduced, which causes the formation of microcells in the plastic structure [126]. Microcells are very small closed gas bubbles that are dispersed inside the plastic. These bubbles are formed by the release of dissolved gas in the plastic during rapid depressurization [127,128]. The structure of a typical miclocellural system is shown in Figure 6.

Microcell molding leads to a structure with lower density, increased stiffness, and better thermal insulation properties compared to standard injection molding. This technique may prove useful in the production of fiber-assisted thermoplastic door panels [129]. The high fiber content can cause difficulties in molding. The use of the microcellular molding technique ensures that carbon fiber-enhanced polypropylene composites achieve high electrical and mechanical performance [130]. MIM technology reduces product weight and energy consumption [131]. MIM products are a frequent subject of research to optimize the process and obtain products with desired properties. The manufacture of ultralight polypropylene foams reinforced with high-strength polytetrafluoroethylene microfibrils was described [132]. The characteristic mofrologi patterns of injection-molded polycarbonate foams were also identified [133]. Subsequently, Chai et al. developed an innovative method combining the MIM method along with in situ fibrillation to produce extremely lightweight and high-strength foams on poly(lactic acid) and polytetrafluoroethylene [134]. The combination of in sita fibrillation and MIM was also proven beneficial for producing lightweight and sound-absorbing PP and PTFE composite foams [135]. Hou et al. used talc as a nucleating agent to prepare foamed PP products. When the talc content is 10% by weight and the part weight loss is 62.1%, the average cell size is 56.5 μm, and the cell density reaches 1.54 × 107 cells/cm^3^ [136]. Composites obtained by the MIM method with the opening of the mold can have a flexural modulus almost 200% higher than that of their unfoamed counterparts [137]. The study of materials processed by microcellular injection molding is of recent importance and is being carried out with the aim of determining many different directions for the application of this method. It is possible to combine the mim method with the in-mold decorating process. A multiphase heat transfer model with fluid–solid coupling was developed to study the temic response in the combined process [138]. Similar studies were also conducted by Yang et al., which proved that the temperature range has a significant effect on the formation of defects and the crystallization of moldings [139]. Wang et al., in turn, confirmed that the development of microcellular foam in a molded molding leads to significant advances in tensile strength from 44.1 MJ/m^3^/(g·cm^−3^) and 111% of neat LCBPP foams to 172.1 MJ/m^3^/(g·cm^−3^) and 440% of LCBPP/MD blend foams in terms of specific breaking energy and elongation at break, respectively [140]. The MIM technique also works well for processing polyetherether ketone (PEEK), which is widely used in aviation. Optimization of the MIM process yields microcellular PEEK with a weight reduction factor of 17.29% and a tensile strength of 74.13 MPa [141]. Reinforced and lightweight foams obtained by the MIM method were also analyzed in the work by Liu et al. [142] Lee et al. [143], and Yu et al. [144]. Liu et al. studied lightweight and strong basalt fiber-reinforced composite foams. They obtained materials with a cell size of 36.38 μm and increased cell density [142]. In turn, Lee et al. proved that by decreasing the degree of supersaturation, the activation energy of cell nucleation increases. There is a decrease in the nucleation rate, and thus, vesicle formation is reduced [143]. On the other hand, Yu et al. undertook research related to reducing the occurrence of internal defects. The use of microcellular injection molding technology made it possible to reduce deformation and deformation of the products [144].

### 3.4. Variable Mold Temperature Technologies

Injection molding is a widely used process for manufacturing plastic products. Many parameters of the process determine its efficiency, effectiveness, and also the final properties of the product. One of the most important aspects is temperature control and distribution. High temperature is necessary to melt the polymer, while mold cooling is crucial in curing the finished product. Variable mold temperature technologies include systems that allow temperature control during the injection molding process. These parameters are optimized over the years and adjusted to meet specific production and material molding requirements. One well-known method is heating the mold with water under high pressure. Newly developed systems can provide temperatures up to about 180–200 °C. However, it is important to properly develop pipe connections that provide the appropriate flow rate of pressurized hot water [145]. Next, hot oil can be used in mold heating systems. In this case, mold temperatures can reach up to 300 °C with high-powered oil heating. However, the disadvantage of hot oil is the low heat transfer coefficient, which has an important effect on reducing the energy efficiency of this medium [146]. Hot steam is also used in heating systems. Steam heating can increase the mold surface temperature from 35 °C to 135 °C in as little as 11 s [147,148]. Electric heating using a heating plate or heating tube system is also known [149]. Chen et al. proposed electromagnetic induction heating combined with water cooling. The proposed system made it possible to obtain moldings with smaller irregularities and improved roughness by 80% [150]. A new temperature concept with segmented heating ceramics was then developed. The goal of the research was to optimize the process to ensure that a product with locally different properties was obtained using temperature control [151]. Cooling channels produced by 3D printing technology were also designed. Extended cooling systems can reduce the cooling time by up to 30% [152]. Variable mold temperature technologies and the effects of different parameters were also studied by Ruzbarsky et al. [153] and Zhang et al. [154]. In order to optimize the process, Ruzbarsky analyzed four parameters, such as compression time, mold temperature, melt temperate, and pressure. Statistical analysis showed that the most significant parameter is the melt temperature, followed by mold temperature, compression time, and pressure [153]. Zhang et al., in turn, studied the effect of changing the high temperature of the mold cavity wall on the internal morphology of the plastic [154].

### 3.5. Microinjection Molding

The demand in the production of precision microdetails influenced the emergence of microinjection molding (µIM) technolgoies. Miniaturization of plastic parts advanced significantly in recent years through continuous work on process optimization (µIM) [155,156]. The characteristics of this process are low manufacturing costs, short process times, and the ability to produce small-sized parts with sufficiently high precision. The processing parameters of the microinjection molding process are represented in Table 2.

**Figure 7:**
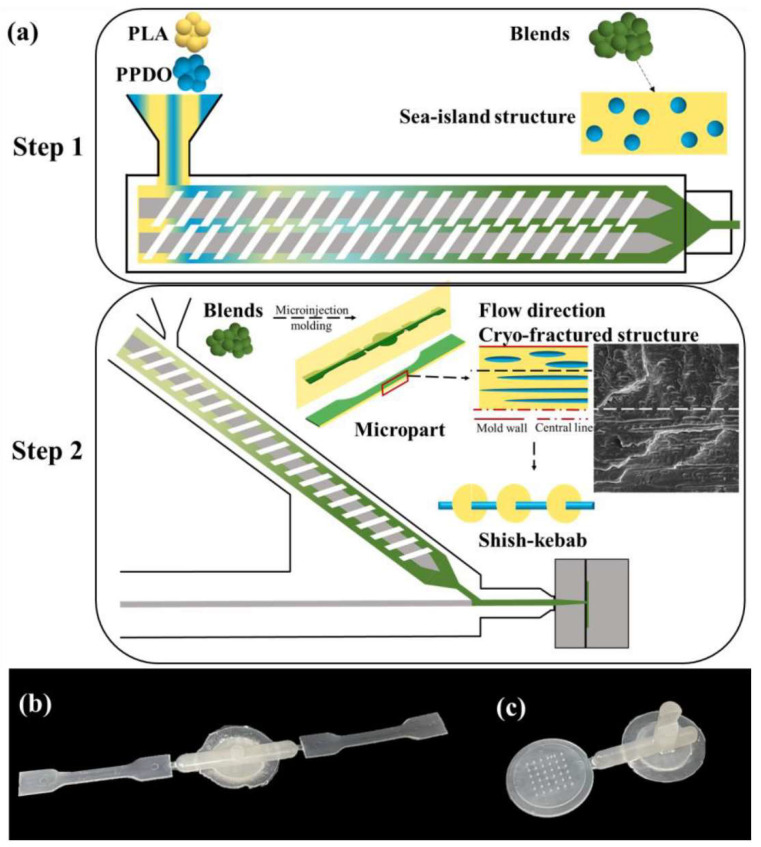
The schematic diagram for preparation of the PLLA/PPDO blend microparts (**a**), the digital picture of the microtensile sample (**b**), and the microneedle array (**c**) [162].

Research on the microforming process is developing at a very intensive pace, leading to the gradual elimination of the limitations of this process. In the process of injection microforming, thermoplastic materials are mostly used, and thermosetting materials are used to a lesser extent [157,158]. However, it should be mentioned that when thermoplastics are used, mostly those with low viscosity are used. The products obtained by microforming usually have good dimensional tolerances. In addition, in most cases, there is no need for finishing work. However, it is very important to properly design the part you want to obtain. Among other things, it is necessary to take into account the appropriate inclination of the walls so that the product can be easily removed from the mold. Equally important is the limitation of sharp corners, which can lead to microtensioning. A problem associated with microfabricated details can be their further assembly into larger systems. Low-temperature bonding as well as ultrasonic welding can be used [159,160,161]. Microinjection molding is widely used in the production of medical devices. The technique is used to produce poly(lactic acid)-based microneedle systems as shown in Figure 7 [162].

Specialized microinjection molds were designed to produce biodegradable microneedles with a drug delivery channel [163]. Subsequently, an attempt was made to process polydioxanone for medical devices such as sutures, stents, and also small implants. Studies showed that the use of the microinjection method for PDO processing makes it possible to obtain samples with high uniformity and shape stability [164]. The technique was also proven excellent in the high-precision and high-performance fabrication of small-module plastic gears [165]. Subsequently, Lin et al. used µIM to fabricate light guiding plates (LGPs) as parts of liquid crystal displays [166]. A limitation of microforming is the occurrence of a frozen layer due to the rapid cooling of the fusible material when it contacts the surface of the low-temperature cavity. A solution to this problem was described by Uyen et al., who proposed an internal gas-assisted mold temperature control system along with a pulsed cooling system. It was proven that the filling capacity of the composite material increases from 65.4% to 100% with local heating of the cavity area (gas temperature pattern from 200 to 400 °C with a 20 s heating cycle) [167]. Next, uneven cavity temperature distribution can be a problem when using thin electric heaters. Optimizing the heater’s performance is achieved by using a transition layer with high thermal conductivity, which provides increased uniformity of cavity temperature distribution [168]. The use of ultrasound was also proposed to affect the plasticization rate of polypropylene rods. As the amplitude of ultrasound increases, it is possible to increase the plasticization rate of PP rods from 67.25 mg/s to 192.41 mg/s [169]. Satisfactory results can also be obtained using variotherm. This treatment makes it possible to reduce the residual stresses while maintaining the accuracy and surface quality of the obtained products [170]. In turn, reducing the thickness of the cavity walls has a significant effect on the rheological behavior of the melt and the flow rate with the observation of strong pressure drops [171]. The processing of isotactic polypropylene filled with β-nucleating agent was also checked. It was shown that as the content of the nucleating agent increases, the shear layers gradually thicken, which translates into improved mechanical properties, especially tensile strength [172].

### 3.6. Rapid Thermal Cycling Molding

**Figure 8:**
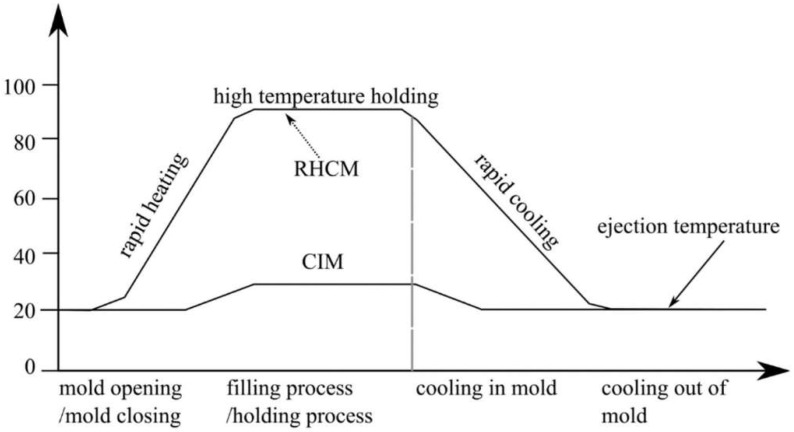
The cycle in the rapid heating cycle molding (RHCM) process (the Y-axis referred to the percentage increase in temperature values, and the X-axis referred to the stages of the injection process) [178].

The rapid thermal cycle molding method is a technique that involves rapid heating and cooling of the mold. This method provides improved quality and efficiency in the molding process. The benefit is to achieve a molded part with very good aspect quality with high gloss without defects, such as joint lines and collapses. With rapid thermal cycling in mind, the mold is heated quickly to the thermal deformation temperature of the polymer before the melt is filled, and then cooled after the packing step [173]. The RTCM method is gaining great importance in modern processing techniques. During the injection molding process, we can distinguish five basic phases, such as melting, filling, packing, cooling, and removal from the mold [174]. It is advantageous to maintain a high temperature during the filling and packaging stages. Such treatment is aimed at providing optimal conditions for the melt polymer to flow, which will favorably affect the repeatability of the molded product [175,176]. In turn, rapid cooling of the finished product can be provided by low temperature. In view of this, methods of rapid cyclic heating and cooling of the mold can improve the quality of the products obtained [177]. Rapid heating cycle molding is presented in Figure 8 [178].

The analysis of RTCM injection molding technology is the subject of many research works. The interest is not only in the optimization of process parameters, but also in the investigation of the mechanisms occurring during plastic processing. The molecular mechanism of replication ability for conventional injection molding and RTCM was presented by Zhang et al. It was proved that the high mold temperature in the RTCM technique ensures that the high teperature is maintained for a long time and Brownian movements are very active in this case. The melt flow of the polymer is crucially related to the mold temperature and the pressure there [179]. Another significant aspect in the RTCM technique is the typical disadvantage of crystalline parts, which is warpage. Multivariate models were developed to predict warpage of the same thickness in molded products [180]. Microporous surface defects, on the other hand, can be eliminated by using electric heating and water cooling controlled over a wide range [181]. A new coating was also proposed to serve as thin-film power heaters that allow rapid heating of the mold cavity above the glass transition temperature of the polymer. To this end, they developed a continuous and dense coating of graphene bonded to carbides on a silicon insert. When the voltage was 240 V, the coating was heated up to 145.6 °C in a short period of 10 s; that is, the average and transient heating rates were able to be as high as 11.6 °C/s and 16.1 °C/s, respectively. The mechanical properties of the resulting products were improved by 37.77% in tensile strength and 256.11% in elongation at yield with a significant reduction in energy consumption [182]. Subsequently, it is possible to use a porous insert to ensure maximum heat transfer between the water and the cavity surface without compromising the structural integrity of the mold [183]. Importantly, the heating efficiency with steam heating can be effectively improved by increasing the thermal conductivity of the cavity and core material, but the situation is completely reversed with electric heating. Therefore, it is also important to optimize mold design methods for RTCM for steam and electric heating, respectively [184].

### 3.7. Multicomponent Injection Molding

Multicomponent injection molding is an advanced plastic injection molding method that allows different materials to be combined in one simultaneous molding process. This technique allows you to create products with different colors, chemical properties, hardness, textures, and other characteristics. The main technical challenges are the selection of process parameters that allow simultaneous processing of two or more components. Equally important is equipping the injection machine with several injection units [185,186]. Multicomponent injection molding was addressed by Park et al. The researchers attempted layered molding with a back core and co-injection. The experiments used a co-injection 1800-ton injection molding machine with a maximum injection pressure of 175 MPa and electrical core-back system. Factors affecting molding were analyzed to obtain parameters closely related to injection molding technology. Relevant numerical simulations were also carried out. The simulations, together with experimental results, confirm the feasibility of multicomponent injection molding by the chosen method [187]. Subsequently, Farias et al. proposed a resin transfer molding (RTM) method for obtaining multicomponent nanocomposites reinforced with carbon fiber and carbon nanotubes. In addition, the epoxy resin was modified with silsesquioxane oligomers. As a result, the selected method allowed for obtaining nanocomposites with a tensile strength of 303 ± 41 MPa and an impact strength of 1.0 ± 0.3 kJ·m^−1^ [188]. The possibility of economic benefits due to material savings and the possibility of one-step production of a layered product structure were also demonstrated. The use of two-component injection molding indicated the increased quality of the parts obtained as well as the stability of the process described [189]. The use of the multicomponent approach continued developing in recent years in various plastic processing methods and is widely studied in many aspects of the selection of appropriate parameters to eliminate technical problems [190,191,192,193].

### 3.8. Metal Injection Molding

**Figure 9:**
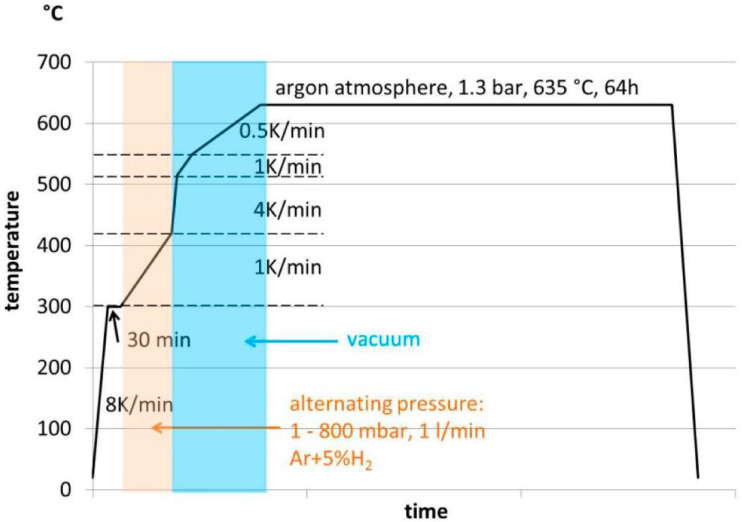
Sintering regime of the magnesium sintering process for MIM Mg-0.9Ca parts [204].

Metal injection molding is an advanced manufacturing technique that allows the molding of metal parts with complex shapes and precise dimensions using a process similar to traditional plastic injection molding [194,195]. Taking a rheological approach, the feedstock for metal-IM technology is a suspension. This fluid consists of suspended metal particles and a continuous phase referred to as a binding system [196]. This system usually consists of various types of polymers, waxes, and other additives that enable metal powder processing. An extremely important parameter is the viscosity of such a system [197,198]. The development of a binder based on superalloy Inconel-718 was handled by Royer et al. A combination with poly ethylene glycol (PEG) and polymers of biological origin, such as polyhydroxyalkanoates (PHA) and Poly(3-hydroxybutyrate-co-3-hydroxyvalerate) (PHBV), was proposed. The solution was to obtain an environmentally friendly binder. The results confirm that the polymer of natural origin PHBV can successfully replace polypropylene, maintaining similar rheological properties and better maximum volume fraction of metallic powder [199]. The analysis of the optimal binder formulation was also addressed by Ibrahim et al. [200] and Yang et al. [201]. Then, using metal-IM technology, a porous titanium filter media was obtained. It was shown that the microporous structure of the interface was the same as that of the parent material, and the porosity of the material reached 32.28% [202]. The Taguchi design method was used by Lin et al. Metal processing parameters were analyzed with the aim of minimizing black lines on the surface of the molded products, which were orthodontic brackets. They ranked the control factors in terms of decreasing effect on powder particle concentration distribution: filling time > melt temperature > packing pressure > mold temperature > gate size [203]. Studies on the processing of magnesium alloys in targeted biomedical applications were also presented. It was demonstrated that magnesium can be sintered into dense parts, providing mechanical properties equivalent to cast materials (Figure 9). Tensile strength of 142 MPa, yield strength of 67 MPa, elastic modulus of 40 GPa, and 8% elongation were demonstrated [204].

### 3.9. Reaction Injection Molding (RIM)

**Figure 10:**
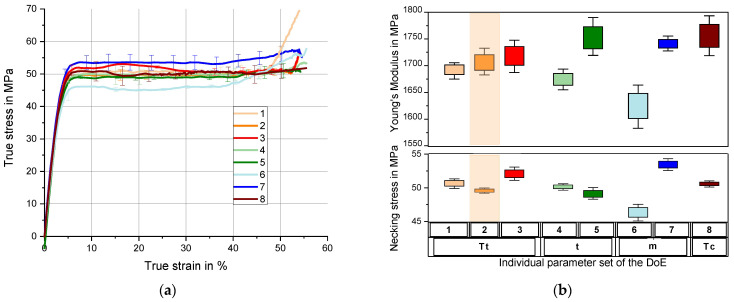
Uniaxial tension tests; (**a**) true stress–strain behavior dependent on different process parameters; (**b**) Young’s modulus and necking stress dependent on process parameters (reference in orange) [211].

Reaction injection molding (RIM) is a technology for producing plastic parts by reactive in situ polymerization. The process uses two or more liquid monomers. The technique is often used; however, its limitation and main challenge is the effective control of mixing between the injected liquid monomers [205,206,207]. Gomes et al. proposed differential static pressures in the injected monomers to control their mixing. The mechanical properties of the injected parts were evaluated. They proved that this parameter correlates with the properties of the molded part and can be used to obtain materials with desired properties [208]. The RIM technique was also proposed for obtaining polyurethane foam with complex geometries. In this case, Seo et al. developed a theoretical model including chemical reactions, foaming, and mold filling. The energy balance equation was analyzed and a three-dimensional numerical simulation was carried out based on the resulting model [209]. The process of manufacturing polyurethane foams using the RIM method was also addressed by Yacoub et al. They defined the basic problems of the process and then conducted multivariate statistical analyses based on principal component analysis (PCA) and projection to latent structure (PLS) models. The use of these correlated processes made it possible to reveal the mechanism of the process and the source of the processing problems [210]. The effect of the RIM process on the thermomechanical properties of polyuratan was studied by Lehmenkuhler et al. The study analyzed the mutual influence of various parameters and their overall significance in the behavior of the material. It was shown that a 20 °C increase in mold temperature can increase Young’s modulus by 2% and constriction stress by 3%. In addition, increasing mass flow and temperature improves Young’s modulus (Figure 10) [211].

A summary of references relating to selected injection processing methods is presented in Table 3.

A summary of the presented injection molding technologies is presented in Table 4. The table contains information on the main features of individual processes, their advantages and disadvantages, and the branches of industry.

There are many applications of WAIM technology, which is especially recommended for the injection of pipe and pipe-like elements. The developing technology allows the use of this method in the production of increasingly complex elements, also branched. Where there is a need for thin-wall moldings, this method is willingly used. GAIM is a method similar in principle to WAIM, but it allows for making a much wider range of moldings without great shape limitations. It is widely used to improve the quality of moldings (air traps, flow lines, and burns). Its great advantage is the possibility of reducing the weight of injected details by gas injection, which results in saving granulate. It can also be used in other applications to reduce their density, e.g., for the production of oars.

Microcellular injection molding is another modern plastic processing technology. The low specific weight of the moldings and significantly improved mechanical properties allow this injection method to be widely used in the aviation industry. MIM can be considered as a new process that combines conventional injection molding with microcellular foaming. The process is considered environmentally friendly, energy-saving, and allows the production of lightweight foamed parts with complex geometries. The multitude of additional technologies that can be combined with MIM increases the scale of possible applications in many applications and indicates a great development potential.

A very intensively developing branch of plastics processing is the MCIM multicomponent injection molding with its various variants. It gives the possibility of producing details in various colors with different textures and, above all, from materials with different mechanical properties. The joining of materials takes place during one production cycle. It is very common to find a combination of a thermoplastic with one or more elastomer, such as in various types of protectors where the core is hard and the carcass, i.e., the protective element, is made of a softer material.

The use of forms made in the RTCM technology is becoming more and more popular, especially in the automotive industry in the production of high gloss details. In particular, it concerns the production of seat elements, headrests, dashboards, and mirrors. The greatest benefit of using this technology is the ability to obtain moldings without the typical disadvantages of other injection technologies, such as dips, hoists, joining lines, and the ability to obtain a high-gloss aspect surface of the detail without the need for additional treatment in the form of varnishing.

There are many methods of multicomponent injection, such as on-mold injection with a rotating half-mold, with a rotary core, and you can also inject a second and third elastomer after transferring the element from the first mold cavity to the second using a robot. The possibilities offered by this technology are very large and are used in the production of automotive components, sports equipment, as well as for the production of masks or diving glasses. The application possibilities are practically unlimited when we need to obtain an element consisting of two, three, or more different plastics. It is most often used for the production of details with a hard matrix of thermoplastics such as PP or PA with additional elastomers or to obtain multicolored parts directly from injection molding.

Currently, MIM is not as widespread as the previously presented injection technologies, but its market share is constantly growing. It is used in the production of complex small elements whose serial production in the classic version is much more expensive or even impossible to achieve. The RIM method and its derivatives, such as PU-RIM, are used to produce thin-walled elements that, thanks to the technology used, can be produced on smaller machines, which contributes to reducing production costs.

## 4. Environmental Impact of Injection Molding Method

**Figure 11:**
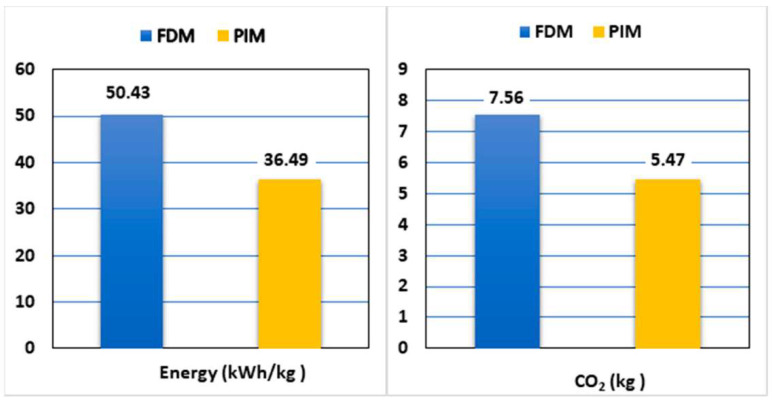
Carbon emission from the FDM and PIM processes [224].

The environmental impact assessment is a process to identify, predict, and mitigate the environmental effects of biophysical process proposals. Injection molding technology, similar to any other processing method, affects the environment. An important parameter in the environmental assessment is the infrastructure of industrial factories, disposal of the resulting waste, and electricity consumption [212,213,214]. The process of injection molding technology involves the production of byproduct waste; for example, in the form of defective products, plastic residues, or used molds. In addition, various chemicals may be used during injection molding to impart desired properties to plastics. Such modifications and use of toxic chemicals are also environmental hazards. In addition, the injection molding process leads to the consumption of a large amount of raw materials, which can lead to the depletion of certain resources and a burden on the environment. Next, injection molding processes are energy intensive, and energy sources often lead to excessive production of greenhouse gases and air pollution [215,216]. In order to minimize the negative environmental impact of injection molding, sustainable manufacturing practices can be employed, such as the use of renewable materials, efficient energy management, optimization of production processes, and the use of recycling and waste recovery. In addition, pursuing clean production and manufacturing in an eco-friendly manner is very beneficial to reduce environmental burdens. An important aspect is to conduct life cycle analysis for all products and planned processes. Evaluating the production processes of injection molding technologies can enable planning and prevention of negative environmental impacts of the process [217,218]. Life cycle assessment (LCA) is used to determine the environmental impact of products, but also of services or processes. The results obtained in the LCA are analyzed to identify areas where special measures should be taken [219,220]. Environmental analysis of the impact of modern processing technologies can be carried out in a number of ways. One of the most important is to determine the energy consumption of a process. Plastics are one of the most widely used materials; therefore, analysis of their safe processing is extremely important [221]. Electricity demand in injection molding processes was studied by various authors. Muller et al. analyzed the injection molding process using double the energy with the goal of increasing process efficiency. Subsequently, Mak et al. pointed to a 20% reduction in energy consumption by reducing the use of petrochemical polymers and using a gas-assisted injection molding process [222]. The idea of life cycle engineering was also introduced by Luchetta et al., who focused on minimizing material consumption in processing while increasing the amount of recycled materials. Monitoring and controlling energy consumption to reduce negative environmental impacts was also studied for other processing processes. It was also proven that electricity consumption can be successfully used as a criterion for assessing the impact of a process, since it causes some of the highest environmental burdens in the entire processing process [223]. Next, the energy demand and carbon dioxide emissions for producing samples from polylactide were analyzed using two methods. The first was additive molding (FDM—Table 5) and the second was injection molding (PIM—Table 6). Functional unit measurement models were developed that can be successfully used to predict energy consumption and carbon emissions in scaled FDM and PIM productions. This can lead to the determination of sustainable PLA production parameters (Figure 11) [224]. 

PLA injection molding was proven to use about 38.2% less energy than FDM technology. In addition, less carbon dioxide is generated per kilogram of PLA molded into the final product compared to additive technology [224].

## 5. Conclusions and Perspectives

Research related to injection molding focuses on understanding and improving the process itself, aiming to achieve higher quality and precision of manufactured products while reducing cycle times and lowering production costs. In this context, researchers are studying the various materials used in the injection molding process, such as plastics, elastomers, and composites, in search of new, more efficient, and environmentally friendly raw materials that can be adapted to specific applications. An important aspect of the research is identifying key parameters of the injection molding process and analyzing their impact on the quality of the final products. This makes it possible to optimize the settings of injection molding machines, leading to better mechanical properties, durability, and aesthetics of products. Researchers also use numerical models and computer simulations to better understand the complex phenomena occurring in the injection molding process. This approach enables faster testing of different scenarios and prediction of results, leading to more efficient solutions. In addition, the research focuses on developing and evaluating new technologies related to injection molding. This includes the exploration of new types of injection molding machines, innovative molding techniques, the search for new materials, and the implementation of intelligent quality control systems that contribute to the progress and development of the field.

Injection molding currently has promising growth prospects in the industry, as automation and robotization of injection molding processes are becoming more advanced. This is leading to increased productivity, precision, and production repeatability, while reducing operating costs. In addition, the introduction of new materials and technologies is opening up new opportunities for injection molding. Examples include higher-strength materials, which are finding applications in the automotive and aerospace industries, and biodegradable plastics, which are responding to the growing demand for greener solutions. Today’s consumers increasingly expect personalized products, and injection molding enables flexibility in design and production, allowing products to be easily customized to meet individual customer needs and preferences. In addition, injection molding plays an important role in sustainable development by reducing raw material consumption, minimizing waste, and promoting recycling. In the medical industry, injection molding is extremely important in the production of medical devices, medical packaging, laboratory instruments, and others. Due to the growing demand for advanced medical technologies, this field is expected to continue to grow, which will contribute to the further development of injection molding. In conclusion, the outlook for injection molding in the industry is promising. Technology development, process flexibility, sustainability, and the growing demand for personalized solutions are all contributing to the growing popularity of this manufacturing method.

## Figures and Tables

**Table 1:** The processing parameters and the processing parameters’ values for the GAIM method [117].

Parameters	Low Level	High Level
Melt injection time	1 s	3 s
Gas delay time	1 s	3 s
Melt temperature	220 °C	240 °C
Mold temperature	50 °C	70 °C
Gas injection pressure	5 MPa	7 MPa

**Table 2:** Processing parameters of the microinjection molding process [156].

Processing Parameters	Material	
	PP	PMMA
Shot size (mm)	10	10
Nozzle temperature (°C)	230	230
Injection velocity (mm/s)	158	158
Packing pressure (MPa)	130	130
Packing time (s)	3	3
Mold temperature (°C)	80	80
Cooling time (s)	30	25

**Table 3:** Summary of the discussed injection molding methods references.

Injection Molding Method	References
Water-assisted injection molding	[97,98,99,100,101,102,103,104,105,106,107,108,109,110,111]
Gas-assisted injection molding	[112,113,114,115,116,117,118,119,120,121,122,123,124,125]
Microcellular injection molding	[126,127,128,129,130,131,132,133,134,135,136,137,138,139,140,141,142,143,144]
Variable mold temperature technologies	[145,146,147,148,149,150,151,152,153,154]
Microinjection molding	[155,156,157,158,159,160,161,162,163,164,165,166,167,168,169,170,171,172]
Rapid thermal cycling molding	[173,174,175,176,177,178,179,180,181,182,183,184]
Multicomponent injection molding	[185,186,187,188,189,190,191,192,193]
Metal injection molding	[194,195,196,197,198,199,200,201,202,203,204]
Reaction injection molding	[205,206,207,208,209,210,211]

**Table 4:** Summary of the properties of the discussed methods.

Method	Disadventage	Adventage	Application
WAIM	Not for multicavity molds and transparent parts.	Thiner walls, better surface quality.	Pipes, chers, and rattan baskets.
GAIM	Complicated rheology model and mold.	Reduce warpage, lower clamping force, thin wall. Reduce cost of raw material.	Lightwidth products.
Microcellular IM	Aspect	Reduce mass and material consumption, enviromental friendly.	Aviation, automotive, and medical industries.
VMTT	Cost of equpment, longe cycle time.	Very good quality product.	Automotive, households.
MM	Parting line and degating issue.	Able to produce 0.1 g components.	Microbearings and pistons, biodegradable implants, endoscopics, and surgery.
RTCM	Long cycle time.	Good quality.	High gloss elements.
Multicomponent	Complicated mold and injection machine.	Multicolor and multifunction elements.	Swimming googles, protectors, elements for the car body, table tennis racket
Metal IM	High initial investment, size of parts.	Wide range of complicated shapes is able to produce.	commercial, medical, dental, and firearms industry.
RIM	Slow cycle time, expensive row matelials.	High density surface on low density core.	Automotive bumpers, spoilers, and fenders.

**Table 5:** FDM process parameters [224].

Parameter	Specification
Nozzle diameter	0.4 mm
Outer shell speed	15 mm/s
100% infill speed	50 mm/s
Speed without extrusion	80 mm/s
Material flow rate	2.5 mm^3^/s

**Table 6:** PIM process parameters [224].

Factor	Level
Injection pressure	25.5 MPa
Nozzle temperature	185 °C
Barrel temperature	176.7 °C
Plate temperature	121.1 °C
Injection time	11 s

## Data Availability

Not applicable.
